# Prospective multicenter evaluation of adherence to the Dutch guideline for children aged 0–16 years with fever without a source—febrile illness in children (FINCH) study

**DOI:** 10.1007/s00431-024-05553-z

**Published:** 2024-04-15

**Authors:** Maya W. Keuning, Nikki N. Klarenbeek, Hidde J. Bout, Amber Broer, Melvin Draaijer, Jeroen Hol, Nina Hollander, Marieke Merelle, Amara Nassar-Sheikh Rashid, Charlotte Nusman, Emma Oostenbroek, Milan L. Ridderikhof, Manouck Roelofs, Ellen van Rossem, Sophie R. D. van der Schoor, Sarah M. Schouten, Pieter Taselaar, Koen Vasse, Anne-Marie van Wermeskerken, Julia M. J. van der Zande, Roy Zuurbier, Merijn W. Bijlsma, Dasja Pajkrt, Frans B. Plötz

**Affiliations:** 1 Amsterdam UMC, Department of Pediatrics, University of Amsterdam, Emma Children’s Hospital, Amsterdam Reproduction and Development Research Institute, Meibergdreef 9, 1105 AZ Amsterdam, The Netherlands; 2 Department of Pediatrics, Tergooi MC, Blaricum, The Netherlands; 3 Department of Pediatrics, Spaarne Hospital, Hoofddorp, The Netherlands; 4 Department of Pediatrics, Noordwest Ziekenhuisgroep, Alkmaar, The Netherlands; 5 Department of Pediatrics, Flevoziekenhuis, Almere, The Netherlands; 6 Department of Pediatrics, Zaans Medical Center, Zaandam, The Netherlands; 7 Department of Emergency Medicine, Amsterdam, UMC , University of Amsterdam, Meibergdreef 9, Amsterdam, Netherlands; 8 Department of Pediatrics, Onze Lieve Vrouwe Gasthuis, Amsterdam, The Netherlands

**Keywords:** Fever without a source, Children, Clinical practice guidelines, Guideline adherence

## Abstract

Evaluation of guidelines in actual practice is a crucial step in guideline improvement. A retrospective evaluation of the Dutch guideline for children with fever without an apparent source (FWS) showed 50% adherence in young infants. We prospectively evaluated adherence to the Dutch guideline and its impact on management in current practice. Prospective observational multicenter cross-sectional study, including children 3 days to 16 years old presented for FWS at one of seven emergency departments in participating secondary and tertiary care hospitals in the Netherlands. Adherence to the Dutch FWS guideline, adapted from the National Institute for Health and Care Excellence (NICE) guideline, was evaluated, and patterns in non-adherence and the impact of non-adherence on clinical outcomes and resource use were explored. Adherence to the guideline was 192/370 (52%). Adherence was lowest in patients categorized as high risk for severe infection (72/187, 39%), compared to the low-risk group (64/73, 88%). Differences in adherence were significant between risk categories (*P* < 0.001) but not between age categories. In case of non-adherence, less urinalysis, fewer bacterial cultures (blood, urine, and cerebral spinal fluid), and less empirical antibiotic treatment were performed (*P* < 0.050). Clinical outcomes were not significantly different between the non-adherence and the adherence group, particularly regarding missed severe infections.

*Conclusions*: We found a high non-adherence rate of 48%, which did not lead to unfavorable clinical outcomes. This substantiates the need for a critical reevaluation of the FWS guideline and its indications for bacterial cultures, viral testing, and antibiotic treatment.

**Table:** 

**What is Known:** *• Despite the development of national guidelines, variation in practice is still substantial in the assessment of febrile children to distinguish severe infection from mild self-limiting disease.* *• Previous retrospective research suggests low adherence to national guidelines for febrile children in practice.*	
**What is New:** *• In case of non-adherence to the Dutch national guideline, similar to the National Institute for Health and Care Excellence (NICE) guideline from the United Kingdom, physicians have used fewer resources than the guideline recommended without increasing missed severe infections.*

**Supplementary Information:**

The online version contains supplementary material available at 10.1007/s00431-024-05553-z.

## Introduction

Fever without an apparent source (FWS) is one of the most common reasons for children to visit the Emergency Department (ED*)* [1]. Most cases of FWS are caused by a mild self-limiting infection, while approximately 6–15% is caused by a severe infection requiring immediate treatment [2]. Clinical presentation is often nonspecific in young children, hampering prompt recognition and adequate management. This diagnostic uncertainty in differentiating severe from self-limiting infections, combined with a higher incidence of severe infection in young infants, leads to high use of ED resources increasing the burden for children presenting with FWS. Likewise, healthcare costs increase, with an almost fivefold higher use of ED resources among infants younger than 3 months compared to children older than 6 months [3]. The Dutch Association of Paediatrics published the national guideline “Fever in secondary care setting in children aged 0–16 years” in 2013, aiming to improve early recognition of severe infections without increasing unnecessary diagnostic testing [4]. The Dutch guideline, adapted from the National Institute for Health and Care Excellence (NICE) of the United Kingdom, provides a step-by-step pathway to assess the risk of infection and subsequently recommends diagnostic testing and treatment.

In general, the purpose of guidelines is to support consistent and effective evidence-based health care and improve clinical outcomes. Practice variation in FWS management, however, remains substantial [5]. A multicenter study reported wide variation in prescriptions of broad-spectrum antibiotics in febrile children between European EDs, of which at least half of the participating EDs had implemented the Dutch or NICE guideline [6]. In a study on the impact of FWS guidelines, the availability of a guideline was not associated with reduced direct costs, and some guidelines did not result in the improvement of clinical outcomes [7]. Thus, the presence of a guideline does not guarantee a positive impact on clinical outcomes. This substantiates the need for guideline evaluation to assess its adherence and applicability in current practice. In turn, this knowledge may reduce unwanted practice variation and ineffective use of guidelines or provide targets for guideline improvement. Evaluation of guideline adherence and outcomes in current practice is a crucial step in guideline development. After the implementation of the Dutch guideline, we retrospectively evaluated guideline adherence, measuring low to moderate adherence in children younger than 3 months without the impact of non-adherence on clinical outcomes [8]. This suggests possibilities for safely reducing diagnostics and treatment in children with FWS. Multicenter prospective evaluation of adherence to the national guideline, including all age groups, is needed to corroborate these findings.

Thus, this prospective multicenter study evaluated the adherence to diagnostic and treatment recommendations of the Dutch national guideline for children aged 0–16 years with FWS. As our secondary aims, we investigated patterns in non-adherence and the impact of non-adherence on clinical outcomes and on the use of diagnostic and therapeutic resources.

## Materials and methods

### Study design and participants

This prospective observational multicenter study included children presenting with FWS at the ED in one of seven participating secondary and tertiary care hospitals, organized in the Pediatric Research and Evaluation Network Amsterdam, in the North-West region of the Netherlands from December 2020 to May 2022. Inclusion criteria, directly adopted from the national guideline, were (I) children aged 3 days to 16 years; (II) presenting with FWS, defined as a temperature of ≥ 38.0 °C at home or during ED visit, and (IIA) no evident focus of infection after history and physical examination or (IIB) a clinical presentation not fitting the potential focus according to the treating physician [4]. Exclusion criteria were children with hospital-acquired or post-operative fever and children initially presenting with a typical febrile seizure in case of a clear focus for the fever.

### Setting

The participating hospitals include one academic tertiary care center, five secondary care teaching hospitals, and one secondary care non-teaching hospital. In the Netherlands, most children are seen by a general practitioner first, in some cases by an emergency physician. Subsequently, all children with fever younger than three months or older children without an apparent source or a sick appearance are referred to the ED for evaluation by a pediatrician or pediatric resident. In this region, the average driving time to primary or secondary care settings should be less than 20 min. National ED visits on average range between 75 and 190 visits per 1000 children [9]. Due to COVID-19 measures, the number of ED and general practitioner visits in 2020 was almost 12% less than in 2019 which mostly normalized after the first wave (December 2020) [9, 10].

### FWS guideline definitions

**Fig. 1:**
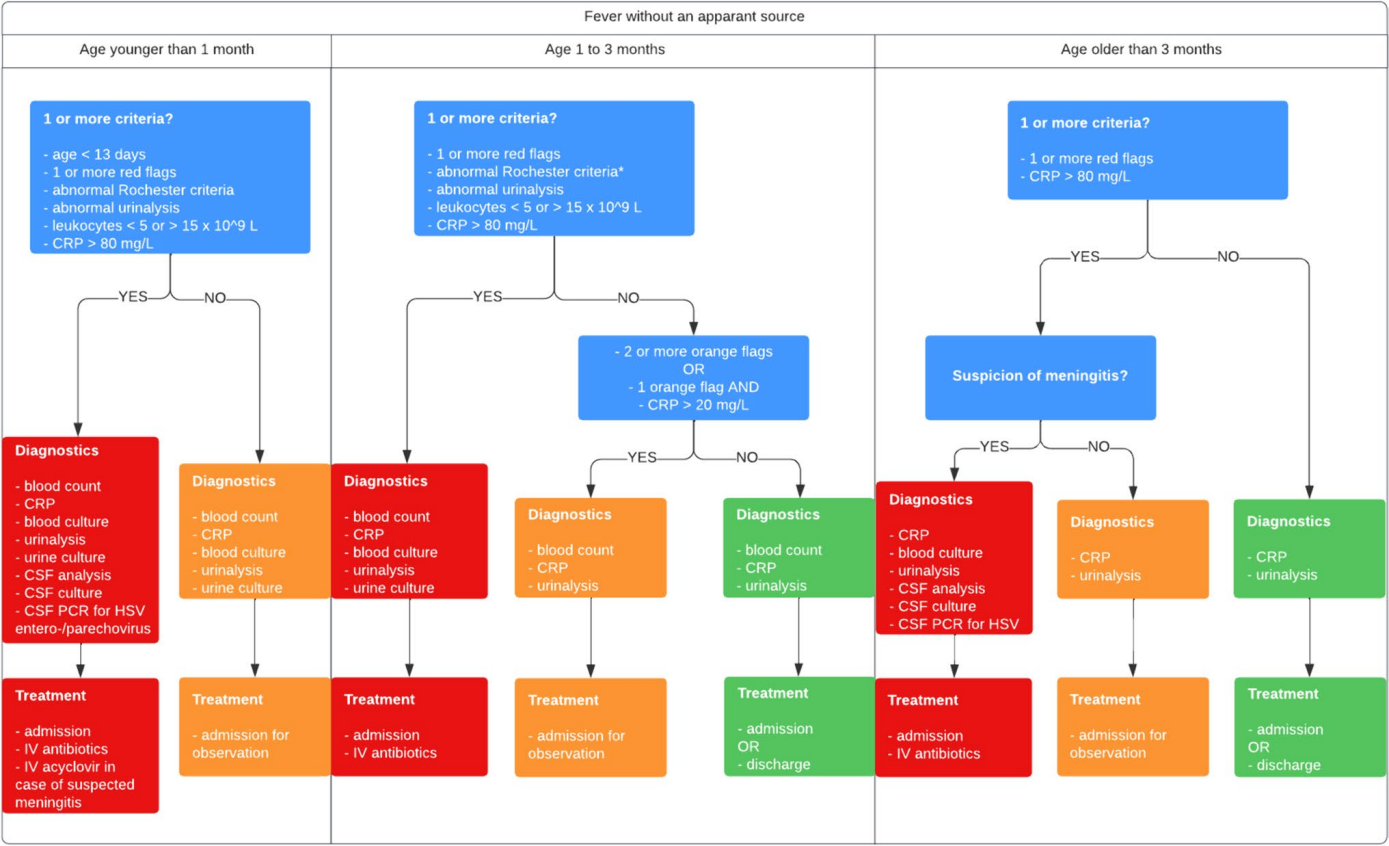
The Dutch national fever without an apparent source guideline recommendations. Pathway for diagnostic and treatment recommendations per age category as defined by the Dutch FWS guideline and derived from the NICE traffic light system. *Rochester criteria is only applicable in children aged younger than 2 months. CSF cerebral spinal fluid, CRP C-reactive protein, HSV herpes simplex virus, IV intravenous, PCR polymerase chain reaction assay

Definitions, specific targeted infections, and age categorization of patients in this study were all in accordance with the national guidelines. Severe infection was defined according to the guidelines as a confirmed Herpes Simplex virus (HSV) encephalitis, bacteriemia, bacterial meningitis, urinary tract infection (UTI), septic arthritis, osteomyelitis, pneumonia, or Kawasaki disease [4]. The guideline recommends separate diagnostic and treatment pathways based on age category of the patient and the risk of severe infection category (Fig. 1). Age categories are defined by the guideline as children younger than 1 month, 1 to 3 months, and older than 3 months, and risk of severe infection is categorized as low (green), intermediate (amber), and high risk (red) (Fig. 1). The risk of severe infection is categorized based on a combination of age, red or amber flags of the NICE traffic light system (supplementary Table 1), Rochester criteria for children younger than 2 months (supplementary Table 2), and the results of initial diagnostic testing.

Initial diagnostic testing includes dipstick urinalysis, C-reactive protein (CRP) and, in children younger than 3 months, white blood cell and differential count (WBC) (Fig. 1). Subsequently, patients younger than 1 month can be categorized as having an intermediate or high risk of severe infection while patients aged 1 to 3 months or older than 3 months can be categorized as having a low, intermediate or high risk of severe infection (Fig. 1). Age younger than 13 days was considered a red flag and therefore always categorized as high risk. Per the age and risk of infection category, the guideline provides recommendations for additional diagnostic testing (bacterial cultures, viral polymerase chain reaction (PCR) testing, cerebral spinal fluid (CSF) analysis, and chest X-rays), hospital admission, and empirical antimicrobial treatment similar to the NICE guideline (Fig. 1). Treatment recommendations include empirical intravenous (IV) antibiotics, oral antibiotics, and IV acyclovir.

When rapid viral testing was positive for influenza or respiratory syncytial virus (RSV) in its endemic season, the guideline states to only perform a diagnostic work-up for a potential severe infection in case of an ill-appearing patient. In the case of a positive rapid viral test and a well-appearing patient, bacterial cultures and empirical antibiotic treatment were not indicated. Since rapid diagnostic testing of severe acute respiratory syndrome coronavirus (SARS-CoV-) 2 was implemented in the course of the study and recommendations for this novel virus were not yet described in the FWS guideline, we calculated adherence for SARS-CoV-2 positive and negative patients separately. Adherence in patients with a positive rapid test for SARS-CoV-2, the coronavirus disease 2019 (COVID-19) cohort, was evaluated similarly to influenza and RSV infection recommendations. For further analyses of patterns and impacts of adherence, patients with a positive SARS-CoV-2 rapid test were excluded.

### Data collection

Eligible patients were managed in the ED according to the judgment of the treating physician. After informed consent was obtained, data regarding the ED visit evaluation and management was collected prospectively by the treating physician. These data included patient characteristics, history and physical examination during ED visit, diagnostic testing and treatment, and testing results and clinical outcomes collected seven days after the initial ED visit. Subsequently, adherence of each case to the Dutch FWS guideline was assessed.

### Data analysis and outcomes

The primary study outcome was the proportion of cases with full adherence to all the recommendations of the guideline. Non-adherence was subdivided into non-adherence to diagnostic and/or treatment recommendations. Cases with an unclear adherence or diagnosis were discussed in the study team blinded for the hospital and treating physician. Antibiotic treatment performed while this was not recommended was also considered non-adherence. In case CRP or urinalysis was missing and the risk category could not be determined, the risk category was considered as missing data and considered as non-adherence.

For the secondary study outcomes, the adherence group and the non-adherence group were compared in terms of patient characteristics, clinical outcomes, and the use of diagnostic and therapeutic resources. For patterns in non-adherence, the patient characteristics were compared between adherence groups. For the clinical outcomes, we assessed potentially missed severe infections based on reported delayed antibiotic treatment (> 12 h) in confirmed bacterial infections, ED revisits, and (re)admissions within 7 days after the initial visit. Further clinical outcomes included final discharge diagnosis as reported in medical charts (see supplementary material for diagnostic criteria), need for IV fluids, O2 support or intensive care unit (ICU) transfer, mortality, length of admission, and delayed antibiotic treatment (> 12 h) overall. The use of resources was measured as the number of performed tests and treatments per age and risk category in the adherence and the non-adherence groups. For reporting of data, a STROBE data reporting checklist can be found in supplementary materials [11].

### Statistical methods

SPSS Statistics version 26.0 (IBM Corp, New York, USA) was used for all analyses. For continuous variables, means with standard deviations or medians with interquartile ranges were calculated. Differences between groups in not-normally distributed variables were analyzed with a Mann–Whitney *U*-test. Categorical variables were depicted in proportions and differences between proportions were analyzed using Pearson’s Fisher’s exact test. The following potential predictors of non-adherence were identified: age category, risk of severe infection category, and comorbidity. As the guideline did not provide a definition of comorbidity, in this analysis, we defined comorbidity as a chronic underlying condition that is expected to last at least one year [12]. All variables with clinical importance and/or a *P* < 0.250 in univariable regression analysis were included in the multivariable regression model after checking for collinearity. For all comparisons, an alpha value of < 0.050 was considered statistically significant. The Bonferroni correction method was used to adjust *p*-values for multiple comparisons. With an expected adherence of 51% based on our previous study, a sample size of *n* = 384 was required to detect a prevalence of adherence to the guideline with a confidence level of 95% and a margin of error of 5% [8].

### Study approval

The study protocol was approved by the Medical Ethics Committee of the Amsterdam University Medical Centers (W20_309 # 20.344), and a waiver for the Medical Research Involving Human Subjects Act was provided. Written informed consent was obtained from parents/guardians and/or children above the legal age of consent.

## Results

### Patient selection

**Fig. 2:**
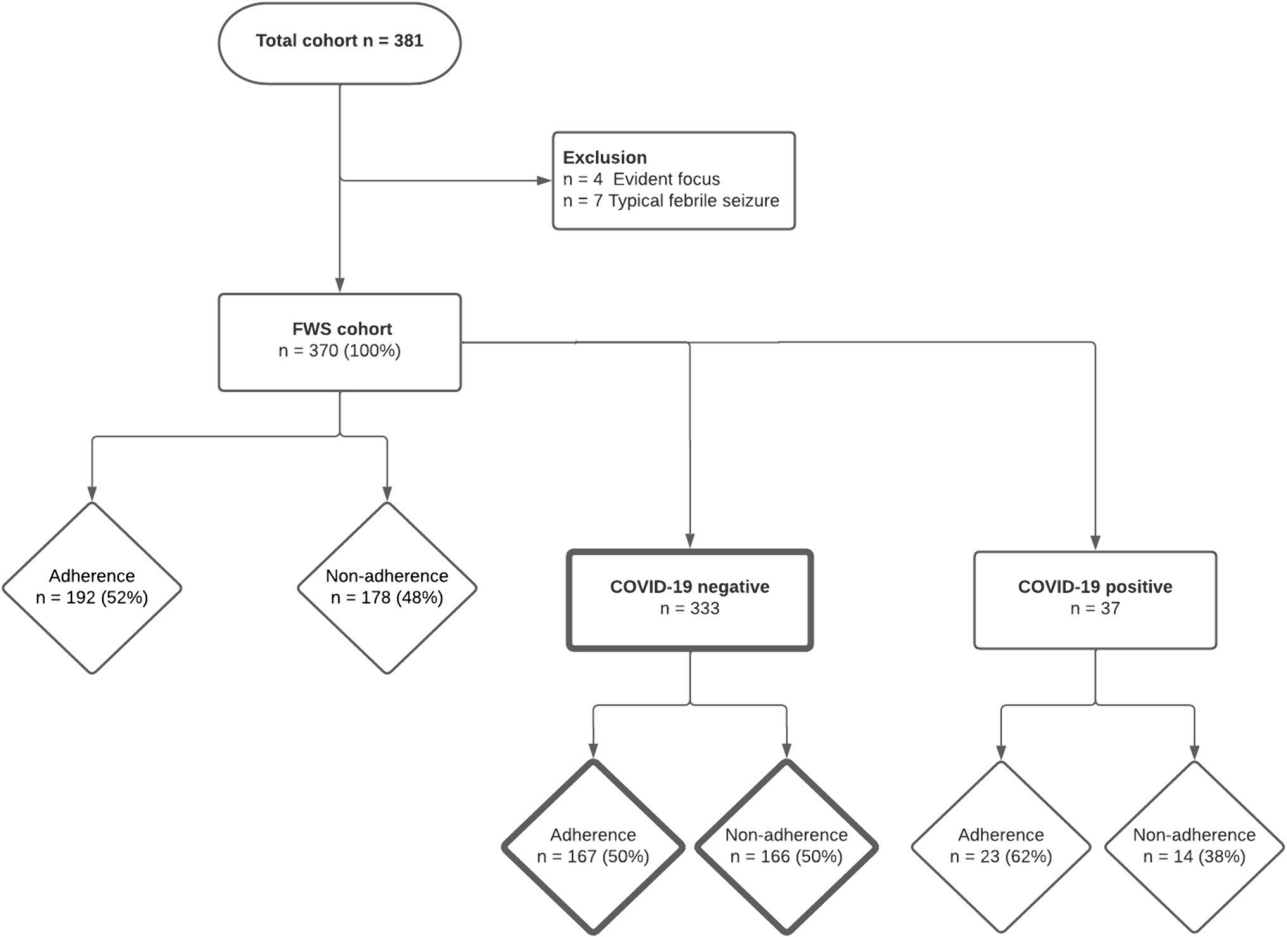
Patient inclusion and adherence. COVID-19 coronavirus disease 2019, FWS, fever without a source, n number

A total of 370 patients were included in the cohort and further analysis of adherence was performed on *n* = 333 after exclusion of SARS-CoV-2 positive patients identified with rapid viral testing upon arrival at the primary ED visit (Fig. 2).

### Patient characteristics (n = 370)

Characteristics, number of performed diagnostic testing and treatment, final diagnoses, and clinical outcomes are shown in Table 1. Most patients were categorized as high risk (201/370, 54%). The overall hospital admission rate was 269/370 (73%) with higher proportions in the high-risk group (169/187, 90%) and medium-risk group (45/57, 80%) compared to low risk (28/73, 38%). Overall, the antibiotic treatment rate was 141/370 (38%), in the high-risk group 115/187 (62%) received antibiotics, in the medium-risk group 16/57 (28%), and 6/73 (8%) in the low-risk group. Of the patients categorized as high risk of severe infection, a lumbar puncture was performed in 88/201 (44%), of which 80/201 (39%) were tested for HSV and 61/201 (30%) patients were empirically treated with IV acyclovir. There were no ICU transfers or deaths in the cohort.

**Table 1:** Patient characteristics of the total FWS cohort and per age category

	**Total cohort**	**Age < 1 month**	**Age 1 to 3 months**	**Age > 3 months**
**Number**	370	110	165	95
**Age in days**	50	15	55	426
Median (IQR)	(24–91)	(9–22)	(37–61)	(243–1095)
**Patient history**				
** Sex, female**	146 (40%)	36 (33%)	73 (44%)	37 (39%)
** Risk of severe infection**				
Low	90 (24%)	-	52 (32%)	36 (39%)
Intermediate	62 (17%)	24 (22%)	11 (7%)	29 (31%)
High	201 (54%)	86 (78%)0	98 (59%)	17 (18%)
Unclear	17 (5%)		4 (2%)	13 (14%)
** Comorbidity**	10 (3%)	0	2 (1%)	8 (8%)
**Vaccination status**				
According to program	75 (20%)	-	-	75 (80%)
Age before start program	275 (74%)	110 (100%)	165 (100%)	-
Not according to program	3 (1%)	-	0	3 (3%)
Unknown	17 (5%)	0	0	17 (17%)
**Time of ED visit**				
06:00–12:00 h	51 (14%)	20 (18%)	21 (13%)	10 (11%)
12:00–18:00 h	115 (31%)	28 (26%)	40 (24%)	47 (50%)
18:00–00:00 h	123 (33%)	30 (27%)	60 (36%)	33 (35%)
00:00–06:00 h	78 (21%)	30 (27%)	43 (26%)	5 (5%)
**Performed diagnostics**				
WBC	335 (91%)	108 (98%)	154 (93%)	73 (77%)
CRP	345 (93%)	108 (98%)	158 (96%)	79 (83%)
Urinalysis	317 (86%)	93 (85%)	143 (87%)	81 (85%)
Blood culture	159 (43%)	76 (69%)	56 (34%)	27 (28%)
Urine culture	183 (50%)	79 (72%)	76 (46%)	28 (30%)
CSF				
- Cells/protein/glucose	82 (22%)	49 (45%)	20 (12%)	13 (14%)
- Culture	89 (24%)	56 (51%)	20 (12%)	13 (14%)
- PCR HSV	81 (22%)	51 (46%)	20 (12%)	10 (11%)
- PCR entero/parechovirus	77 (21%)	49 (45%)	19 (12%)	9 (10%)
Feces				
- Culture	24 (7%)	13 (12%)	6 (4%)	5 (5%)
- PCR entero/parechovirus	94 (25%)	44 (40%)	40 (24%)	10 (11%)
Throat swab				
- PCR entero/parechovirus	35 (10%)	17 (16%)	12 (7%)	6 (6%)
- PCR respiratory viruses	236 (64%)	70 (64%)	113 (69%)	53 (56%)
- PCR SARS-CoV-2	310 (84%)	98 (89%)	142 (86%)	70 (74%)
Chest X-ray	21 (6%)	3 (3%)	0	18 (19%)
**Treatment, N (%)**				
Admission	269 (73%)	107 (97%)	118 (72%)	44 (46%)
Oral antibiotics	20 (5%)	0	7 (4%)	13 (14%)
IV antibiotics	121 (33%)	70 (64%)	32 (19%)	19 (20%)
IV acyclovir	61 (17%)	41 (37%)	15 (9%)	5 (5%)
Discharge and re-evaluation	25 (7%)	1 (1%)	8 (5%)	16 (17%)
**Confirmed diagnosis**				
Bacterial	47 (13%)	20 (18%)	20 (12%)	7 (7%)
Viral	144 (39%)	59 (54%)	64 (39%)	21 (22%)
Bacterial and viral	9 (2%)	2 (2%)	4 (2%)	3 (3%)
Unconfirmed	170 (46%)	29 (26%)	77 (47%)	64 (67%)
**Severe infection**^**a**^				
Bacteriemia	10 (3%)	6 (6%)	2 (1%)	2 (2%)
Bact meningitis	3 (1%)	0	3 (2%)	0
UTI^b^	54 (15%)	22 (20%)	22 (13%)	10 (11%)
Septic arthritis	1 (0.3%)	0	0	1 (1%)
Osteomyelitis	0	0	0	0
Pneumonia	7 (2%)	0	0	7 (7%)
HSV encephalitis	2 (0.5%)	1 (0.9%)	1 (0.6%)	0
Kawasaki disease	0	0	0	0
**Non-severe infection**^**a**^				
Enterovirus	49 (13%)	29 (26%)	19 (12%)	1 (1%)
Parechovirus	11 (3%)	6 (5%)	4 (2%)	1 (1%)
Influenza	16 (4%)	0	14 (8%)	2 (2%)
SARS-CoV-2	37 (10%)	10 (10%)	23 (14%)	4 (4%)
RSV	2 (1%)	1 (1%)	0	1 (1%)
Rhinovirus	27 (7%)	8 (7%)	11 (7%)	8 (8%)
Other viral	8 (2%)	0	3 (2%)	5 (5%)
**Clinical outcomes**				
IV fluids	5 (1%)	1 (1%)	1 (1%)	3 (3%)
Respiratory support	3 (1%)	2 (2%)	0	1 (1%)
ICU transfer	0	0	0	0
Mortality	0	0	0	0
Delayed antibiotics	17 (5%)	5 (5%)	8 (5%)	4 (4%)
Delayed antibiotics in confirmed bacterial infection	8 (2%)	2 (2%)	5 (3%)	1 (1%)
ED revisit	17 (5%)	1 (1%)	7 (4%)	9 (10%)
Readmission	7 (2%)	0	5 (3%)	2 (2%)

*-* not applicable, *CSF* cerebral spinal fluid, *CRP* C-reactive protein, *ED* emergency department, *FWS* fever without a source, *HSV* herpes simplex virus, *ICU* intensive care unit, *IV* intravenous, *IQR* interquartile range, *N* number, *PCR* polymerase chain reaction assay, *RSV* respiratory syncytial virus, *SARS-CoV-2* severe acute respiratory syndrome coronavirus 2, *SD* standard deviation, *sec* seconds, *UTI* urinary tract infection, *WBC* white blood cell count

^a^Severe and non-severe infection as defined by the guideline

^b^7/54 (13%) UTIs not confirmed by culture but probable diagnosis based on elevated CRP levels and positive urinalysis and/or radiological results

### Severe infections

A final diagnosis of bacterial infection was confirmed in 56/370 (15%), a clinical diagnosis of pneumonia in 7/370 (2%) and HSV encephalitis in 2/370 (0.5%) (Table 1). Viral infections were identified in 153/370 (41%). Rapid viral testing identified a viral infection upon presentation in 53/370 (14%). A viral and bacterial coinfection was confirmed in 9/370 (2%). The bacterial infection rate correlated with the risk of severe infection according to the guideline categorization: 2/90 (2%) in the low-risk group compared to 6/62 (10%) in the intermediate and 48/209 (24%) in the high-risk group. Similarly, the bacterial infection rate was higher in patients younger than 1 month compared to the older age categories (Table 1).

### Adherence (n = 370)

Full adherence to all recommendations was reported in 192/370 (52%) and after exclusion of patients with positive SARS-CoV-2 rapid testing 167/333 (50%) (Fig. 2). Non-adherence to one recommendation was 67/370 (18%), to two recommendations was 47/370 (13%), to three separate recommendations was 27/370 (7%), and to four or more recommendations was 39/370 (11%).

### Patterns of non-adherence (n = 333)

**Fig. 3:**
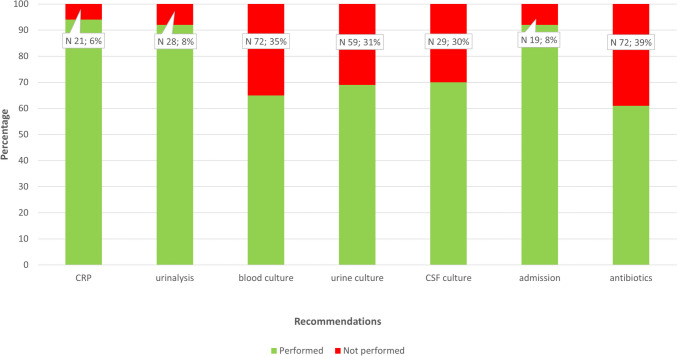
Adherence per recommendation. Proportions of performed diagnostic and therapeutic recommendations (sample *n* = 333), with the total bar representing all patients for which CRP (*n* = 333), urinalysis (*n* = 333), blood culture (*n* = 206), urine culture (*n* = 190), cerebral spinal fluid (CSF) culture (*n* = 98), admission (*n* = 244), or antibiotic treatment (*n* = 187) was recommended according to the FWS guideline. Patients with a positive rapid test for SARS-CoV-2 were excluded from the analysis

**Fig. 4:**
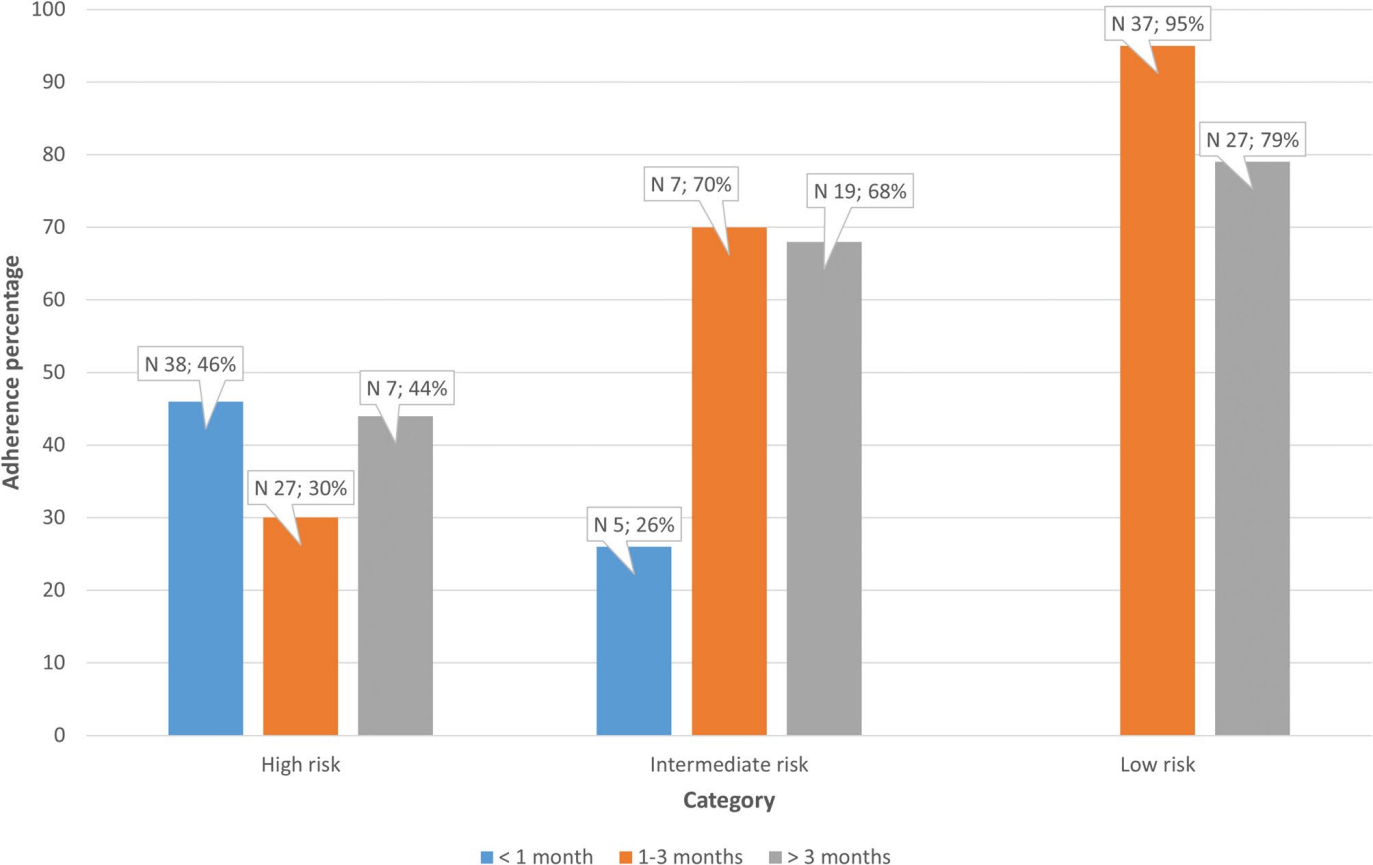
Adherence per age and risk category. Adherence depicted in the bars is shown in proportions per risk category and per age category, as categorized according to the FWS guideline (sample *n* = 333). The number of patients per category was as follows: < 1 month, high risk, *n* = 82; 1–3 months, high risk, *n* = 89; > 3 months, high risk, *n* = 16; < 1 month, intermediate risk, *n* = 19; 1–3 months, intermediate risk, *n* = 10; > 3 months, intermediate risk, *n* = 28; 1–3 months, low risk; *n* = 39; and > 3 months, low risk, *n* = 34. Patients with a positive rapid test for SARS-CoV-2 were excluded from the analysis

For our secondary aim, we evaluated patterns in non-adherence by describing patient characteristics per adherence group, excluding patients with a positive SARS-CoV-2 rapid test. In case of non-adherence, mostly blood or urine cultures and lumbar punctures were not performed (Fig. 3). Antibiotics were not started in 72/187 (39%) of patients in whom empirical treatment was recommended (Fig. 3). Figure 4 shows adherence per age and risk category. Between participating hospitals, adherence ranged from 39 to 63% with characteristics per site reported in supplementary Table 5. Adherence was lowest in patients younger than 1 month categorized as the intermediate risk of severe infection 5/19 (26%) and highest in patients 1 to 3 months categorized as low risk 37/39 (95%). Differences in adherence were significant between risk categories (*P* < 0.001) but not between age categories (*P* = 0.095). In multivariable logistic regression (including age category, risk of severe infection, and comorbidity), only the risk category was an independent predictor for non-adherence: the high-risk category showed an adjusted odds ratio of 11.67 (95% confidence interval 5.18–26.25, *P* < 0.001) compared to the low-risk category. There were no significant differences between the adherence and the non-adherence group in the number of severe infections or time of ED visit (Table 2).

**Table 2:** Characteristics and clinical outcomes in the adherence and non-adherence group

	**Adherence n= 167**	**Non-adherence n= 166**	**P**
**Patient characteristics**			
- Median age	61 days (IQR 26–213)	46 days (IQR 22–76)	0.153
**Time of ED visit**			
- 06:00–12:00 h	19/166 (11%)	27/164 (17%)	NS
- 12:00–18:00 h	54/166 (33%)	53/164 (33%)	
- 18:00–00:00 h	57/166 (34%)	48/164 (29%)	
- 00:00–06:00 h	36/166 (22%)	36/164 (22%)	
**Severe infection risk**			
- Low risk	64/167 (38%)	9/150 (6%)	< 0.001*
- Intermediate risk	31/167 (19%)	26/150 (18%)	
- High risk	72/167 (43%)	115/150 (76%)	
**Confirmed diagnosis**			
- Bacterial	26/167 (16%)	21/166 (13%)	0.219*
- Viral	52/167 (31%)	57/166 (34%)	
- Bacterial and viral	6/167 (4%)	1/166 (1%)	
- Unknown	83/167 (50%)	87/190 (52%)	
**Clinical outcomes**			
- Median admission duration	2 days (IQR 1–4)	1 day (IQR 0–3)	**0.010**
- Readmission	0/166 (1%)	5/166 (3%)	NS
- Delayed antibiotics	7/166 (4%)	8/166 (5%)	NS
- Missed severe infections	3/167 (2%)	5/166 (3%)	NS
- ICU transfer	0	0	NS
- Mortality	0	0	NS

Proportions were compared between the adherence and non-adherence groups with chi-square or Fisher’s exact testing (sample *n* = 333). An alpha value of < 0.050 was considered statistically significant and depicted in bold. Patients with a positive rapid test for SARS-CoV-2 were excluded from the analysis

*ED* emergency department, *ICU* intensive care unit, *IQR* interquartile range, *NS* not significant

^*^Bonferroni-corrected *p*-value

### Impact of non-adherence (n = 333)

To evaluate the impact of non-adherence, we compared the clinical outcomes (Table 2) and the number of performed diagnostic testing and treatment in the adherence group versus the non-adherence group (Tables 3 and 4). The median admission rate was one day shorter in the non-adherence group (*P* = 0.010). There were no significant differences in mortality, ICU admission, readmission rates, or missed severe infections between the adherence and non-adherence groups (Table 2). If treated according to the guideline, 187 patients would have received antibiotic treatment of which 53 patients were diagnosed with a bacterial infection. In the adherence group, 47% of patients without confirmed bacterial infection received antibiotics therapy while 31% in the non-adherence group received antibiotics.

**Table 3:** Differences in testing and treatment for high risk of severe infection

Risk category	High risk for severe infection								
Age category	< 1 month			1 to 3 months			> 3 months		
Characteristics	Adherence (*N* = 38)	Non-adherence (*N* = 44)	*p-*value	Adherence (*N* = 27)	Non-adherence (*N* = 62)	*p-*value	Adherence (*N* = 7)	Non-adherence (*N* = 9)	*p-*value
Diagnostics, *N* (%)									
Blood count	38 (100%)	43 (98%)	1.000	27 (100%)	61 (98%)	1.000	7 (100%)	7 (78%)	0.475
CRP	38 (100%)	43 (98%)	1.000	27 (100%)	62 (100%)	1.000	7 (100%)	7 (78%)	0.475
Urinalysis	38 (100%)	37 (84%)	0.765	27 (100%)	56 (90%)	0.172	7 (100%)	8 (89%)	1.000
Blood culture	**38 (100%)**	**27 (61%)**	**0.001**	**27 (100%)**	**20 (32%)**	**0.001**	**7 (100%)**	**4 (44%)**	**0.034**
Urine culture	**38 (100%)**	**28 (64%)**	**0.001**	**27 (100%)**	**27 (44%)**	**0.001**	6 (86%)	3 (33%)	0.060
CSF culture	**38 (100%)**	**16 (36%)**	**0.001**	**16 (60%)**	**2 (3%)**	**0.001**	7 (100%)	5 (56%)	0.088
CSF PCR entero/parechovirus	**38 (100%)**	**11 (25%)**	**0.001**	**16 (59%)**	**2 (3%)**	**0.001**	**6 (86%)**	**2 (22%)**	**0.041**
Treatment, *N* (%)									
Admission	38 (100%)	42 (96%)	0.497	27 (100%)	50 (81%)	0.099	7 (100%)	6 (67%)	0.212
Antibiotics	**38 (100%)**	**28 (64%)**	**0.001**	**27 (100%)**	**10 (16%)**	**0.001**	7 (100%)	5 (56%)	0.088
Acyclovir	**29 (76%)**	**11 (25%)**	**0.001**	**12 (44%)**	**2 (3%)**	**0.001**	4 (43%)	1 (11%)	0.106

For all children categorized as high risk of infection, the performed diagnostic testing and treatment are depicted per age group and compared between the adherence and non-adherence groups (sample *n* = 187). Proportions were compared between the adherence and non-adherence groups with chi-square or Fisher’s exact testing and corrected for multiple testing. An alpha value of < 0.050 was considered statistically significant and depicted in bold. Patients with a positive rapid test for SARS-CoV-2 were excluded from the analysis

*CRP* C-reactive protein, *N* number, *PCR* polymerase chain reaction

**Table 4:** Differences in testing and treatment for low/intermediate risk of severe infection

Risk category	Low/intermediate risk for severe infection								
Age category	< 1 month			1 to 3 months			> 3 months		
Characteristics	Adherence (*N* = 5)	Non-adherence (*N* = 14)	*p-*value	Adherence (*N* = 44)	Non-adherence (*N* = 5)	*p-*value	Adherence (*N* = 46)	Non-adherence (*N* = 16)	*p-*value
Diagnostics, *N* (%)									
Blood count	5 (100%)	14 (100%)	1.000	41 (93%)	5 (100%)	1.000	41 (89%)	13 (81%)	0.668
CRP	5 (100%)	14 (100%)	1.000	43 (98%)	5 (100%)	1.000	46 (100%)	14 (88%)	0.063
Urinalysis	5 (100%)	12 (86%)	0.591	39 (89%)	5 (100%)	0.644	**44 (96%)**	**11 (69%)**	**0.010**
Blood culture	**5 (100%)**	**3 (21%)**	**0.005**	6 (14%)	1 (20%)	1.000	12 (26%)	2 (13%)	0.322
Urine culture	**5 (100%)**	**5 (36%)**	**0.033**	16 (36%)	3 (60%)	0.363	**16 (35%)**	**1 (6%)**	**0.048**
CSF culture	0 (0%)	0 (0%)	1.000	1 (2%)	0 (0%)	1.000	0 (0%)	0 (0%)	1.000
CSF PCR entero/parechovirus	0 (0%)	0 (0%)	1.000	1 (2%)	0 (0%)	1.000	0 (0%)	0 (0%)	1.000
Treatment, *N* (%)									
Admission	5 (100%)	14 (100%)	1.000	26 (60%)	2 (40%)	0.639	**24 (52%)**	**2 (13%)**	**0.007**
Antibiotics	2 (40%)	0 (0%)	0.058	1 (2%)	0 (0%)	1.000	12 (26%)	7 (44%)	0.218
Acyclovir	0 (0%)	0 (0%)	1.000	0 (0%)	0 (0%)	1.000	0 (0%)	0 (0%)	1.000

For all children categorized as low or intermediate risk of infection, the performed diagnostic testing and treatment are depicted per age group and compared between the adherence and non-adherence groups (sample *n* = 130). Proportions were compared between the adherence and non-adherence groups with chi-square or Fisher’s exact testing and corrected for multiple testing. An alpha value of < 0.050 was considered statistically significant and depicted in bold. Patients with a positive rapid test for SARS-CoV-2 were excluded from the analysis

*CRP* C-reactive protein, *N* number, *PCR* polymerase chain reaction

Regarding missed severe infections, the FWS guideline did not recommend antibiotic treatment in three patients (2%) in the adherence group, who were later diagnosed with a UTI (*n* = 2) or bacterial meningitis. In the non-adherence group, five patients (3%) did not receive antibiotic treatment while this was recommended. These patients received delayed antibiotic treatment and were later diagnosed with UTI (*n* = 3), UTI with bacteremia, or bacterial with viral meningitis. In the high-risk category, there were significantly lower rates of blood/urine cultures, lumbar punctures, and antimicrobial treatment in the non-adherence group (Table 3). In the low-risk category, significantly fewer cultures were performed in patients younger than 1 month and urinalysis and admission in patients older than three months (Table 4).

### Adherence in COVID-19 cohort (n = 37)

In 26/37 (70%) of the COVID-19 cohort, there was no ill appearance reported, and the other 11/37 (30%) presented with an ill appearance, meaning the presence of a red flag. Adherence was 23/37 (62%) in the COVID-19 cohort. Non-adherence in this cohort consisted of bacterial cultures or empirical antibiotic treatment while there was no ill appearance or incomplete bacterial cultures or antibiotic treatment in case of an ill appearance. Hospital admission rate was 24/37 (65%), and IV antibiotic treatment was 4/37 (11%). One patient was diagnosed with Multisystem Inflammatory Syndrome in Children, one co-infection with influenza was reported, and one bacterial coinfection with a UTI.

## Discussion

We found adherence to the Dutch national guideline in half of children presenting with FWS at the ED. Adherence to the guideline was lower in children categorized as high risk of severe infection. In the non-adherence group, significantly fewer urinalysis, bacterial cultures, lumbar punctures, and antimicrobial treatments were performed compared to the adherence group with no differences in missed severe infections.

In our previous retrospective study in infants younger than 3 months, adherence was 51% with fewer cultures and antibiotic treatment performed in case of non-adherence. We were able to corroborate these findings in the current more multicentered and prospectively performed study covering all age groups [8]. Adherence was particularly low in the high-risk groups, as well as the younger age groups, for which the guideline provides more numerous recommendations compared to the older or low-risk patients. While these are the first studies describing adherence to the Dutch guideline, the NICE guideline for FWS similarly showed low adherence across several European EDs in bacterial cultures and antibiotic treatment [13]. Regarding the measurement of NICE-recommended vital signs, a 52% non-adherence was reported [14]. Non-adherence can be explained by several factors concerning the physician’s knowledge, attitudes, and behavior as well as complicated or variating guidelines [15]. A comparison of ten high-income countries showed wide variation between guidelines in definitions of high risk for severe infection [16]. This variation in defining a patient as high risk, and thus indicating cultures and treatment, could play a role in the low adherence in patients categorized as high risk in this study. We deliberately did not include a survey of reasons for non-adherence so as to not affect the behavior of physicians during our evaluation. Particularly in missed severe infections, it is of importance to understand the reasoning behind a physician’s non-adherence. Some patterns of non-adherence could indicate a lack of physician’s awareness which should be targeted for education to improve adherence. For instance, less urinalysis and urine cultures were performed than recommended by the guidelines. Moreover, often urine cultures were not performed after a negative urinalysis while urinalysis of young infants does not have 100% rule-out value for a UTI [17]. As UTIs are the most frequent cause of FWS yet their clinical presentation is remarkably nonspecific, this requires specific attention [18].

Our findings raise the question of whether interventions need to be applied to increase adherence, or if these adherence rates are actually a symptom of decreased applicability of the current FWS guideline or its acceptability for physicians or patients. Physicians did not start antibiotic treatment in the majority of children aged 1 to 3 months categorized as high risk, indicating that physicians applied a higher threshold to antibiotic treatment than the guideline. The presence of one red flag already categorizes as high risk, while in a validation study of the traffic light system, most red flags showed little individual rule-in value for severe infection across multiple datasets [19]. Furthermore, a large meta-analysis calculated roughly half the rate of severe infection in this age group compared to younger infants [20]. The new United States guideline published in 2022 similarly suggested a less defensive approach to well-appearing febrile infants while the 2021 update of the NICE guideline remained to recommend their traffic light system and concurrent recommendations [21, 22]. If all patients were treated according to the guideline, 187 patients would have received antibiotic treatment of which less than a third was diagnosed with a bacterial infection. Importantly, there was no increase in missed severe infections or adverse clinical outcomes in our study due to non-adherence. This substantiates the need for a critical reevaluation of the FWS guideline and its indications for bacterial cultures and treatment. As we reported in our international comparison study, guidelines for febrile children are largely similar across countries and continents, and thus, our results and the need for critical evaluation of guidelines are generalizable to many high-income countries in addition to countries using the NICE guideline [16].

Moreover, rapid viral diagnostic testing, including SARS-CoV-2, RSV, and influenza, revealed a plausible source in 14% of all FWS cases. Although these tests may mostly be of value during their endemic seasons, this illustrates the potential to decrease further bacterial testing and treatment. In line with the low rate of bacterial coinfections in our cohort, other studies showed a significantly lower risk for severe infection in febrile infants positive for viral infections compared with virus-negative infants [23, 24]. As viral PCR testing for enterovirus, although not available as a rapid test, has also shown potential to shorten admission duration and use of antibiotic treatment, evidence-based guidance on viral testing (both rapid and non-rapid methods) should be implemented in the revised FWS guideline [25]. Considering we found a viral cause in our cohort in 41% with low rates of bacterial coinfection, future research efforts in rapid viral testing are needed to decrease ED resource use.

This guideline evaluation study faced several limitations. First, we were not able to register patients presented at the ED who were not recruited by the physician or refused participation. However, considering the distribution of high-risk patients was equal to our previous retrospective sample, the amount of selection bias may therefore be limited [8]. Second, adherence could be overestimated if participation in the study influenced the physician’s behavior. As our primary and secondary outcomes are very comparable to our previous adherence study, which is less vulnerable to participation bias due to its retrospective design, the impact on outcomes may be negligible. Third, our inclusion partly took place during the COVID-19 pandemic, which could have influenced the epidemiology of other pathogens and healthcare-seeking behavior. Fourth, we did not include a random effects model to analyze differences between sites in addition to the adherence per site and site-specific data. Fifth, although methodologically explainable considering participation bias, missing the rationale of physicians for non-adherence is an important limitation in the interpretation of our results.

In conclusion, in our multicenter prospective evaluation of the Dutch guideline for children presenting with FWS the high non-adherence rate did not lead to unfavorable clinical outcomes. In case of non-adherence, physicians have used fewer ED resources than the guideline recommended without increasing missed severe infections. This substantiates the need for a critical reevaluation of the FWS guideline and its indications for bacterial cultures, viral testing, and antibiotic treatment.

### Supplementary Information

Below is the link to the electronic supplementary material.

Supplementary file1 (DOCX 29 KB)

## Data Availability

The datasets generated during and/or analyzed during the current study are available from the corresponding author upon reasonable request.

## Abbreviations

COVID-19: Coronavirus disease 2019
CRP: C-reactive protein
CSF: Cerebral spinal fluid
ED: Emergency department
IV: Intravenous
HSV: Herpes Simplex virus
FWS: Fever without an apparent source
NICE: National Institute for Health and Care Excellence
PCR: Polymerase chain reaction
RSV: Respiratory syncytial virus
SARS-CoV-2: Severe acute respiratory syndrome coronavirus 2
ICU: Intensive care unit
UTI: Urinary tract infection
WBC: White blood cell count
