# Correction to: Prospective multicenter evaluation of adherence to the Dutch guideline for children aged 0–16 years with fever without a source—febrile illness in children (FINCH) study

**DOI:** 10.1007/s00431-024-05594-4

**Published:** 2024-05-06

**Authors:** Maya W. Keuning, Nikki N. Klarenbeek, Hidde J. Bout, Amber Broer, Melvin Draaijer, Jeroen Hol, Nina Hollander, Marieke Merelle, Amara Nassar-Sheikh Rashid, Charlotte Nusman, Emma Oostenbroek, Milan L. Ridderikhof, Manouck Roelofs, Ellen van Rossem, Sophie R. D. van der Schoor, Sarah M. Schouten, Pieter Taselaar, Koen Vasse, Anne-Marie van Wermeskerken, Julia M. J. van der Zande, Roy Zuurbier, Merijn W. Bijlsma, Dasja Pajkrt, Frans B. Plötz

**Affiliations:** 1 Amsterdam UMC, Department of Pediatrics, University of Amsterdam, Emma Children’s Hospital, Amsterdam Reproduction and Development Research Institute, Meibergdreef 9, 1105 AZ Amsterdam, The Netherlands; 2 Department of Pediatrics, Tergooi MC, Blaricum, The Netherlands; 3 Department of Pediatrics, Spaarne Hospital, Hoofddorp, The Netherlands; 4 Department of Pediatrics, Noordwest Ziekenhuisgroep, Alkmaar, The Netherlands; 5 Department of Pediatrics, Flevoziekenhuis, Almere, The Netherlands; 6 Department of Pediatrics, Zaans Medical Center, Zaandam, The Netherlands; 7 Department of Emergency Medicine, UMC, University of Amsterdam, Meibergdreef 9, AmsterdamAmsterdam, Netherlands; 8 Department of Pediatrics, Onze Lieve Vrouwe Gasthuis, Amsterdam, The Netherlands

**Correction to: European Journal of Pediatrics** 10.1007/s00431-024-05553-z

In this published article entitled “Prospective multicenter evaluation of adherence to the Dutch guideline for children aged 0–16 years with fever without a source—febrile illness in children (FINCH) study”, one of the author’s corrections to the author's names in the proof was incorrectly adjusted.

Incorrect: Nassar-Sheikh Nassar-Sheikh Rashid

Correct: Amara Nassar-Sheikh Rashid

(given name: Amara, surname: Nassar-Sheikh Rashid)

The original article has been corrected.

