# Supplementary material for: Prospective multicenter evaluation of adherence to the Dutch guideline for children aged 0–16 years with fever without a source—febrile illness in children (FINCH) study
Source: Eur J Pediatr. 2024 Apr 15;183(7):2921–33. doi: 10.1007/s00431-024-05553-z (PMC11192673; doi:10.1007/s00431-024-05553-z)
Supplement: Supplementary file 1 — Supplementary file1 (DOCX 29 KB) [file 431_2024_5553_MOESM1_ESM.docx]

**Supplementary tables**

|  | Low risk (green) | Intermediate risk (amber) | High risk (red) |
| --- | --- | --- | --- |
| Color | • Normal color | Pallor reported by  parent/carer | Pale/mottled/ashen |
| Activity | • Responds normally to social cues  • Content/smiles  • Stays awake or  awakens quickly  • Strong normal cry or not crying | • Not responding normally  to social cues  • No smile  • Wakes only with  prolonged stimulation  • Decreased activity • Disease course different according to parent/carer • Ill-appearance according to physician | • No response to social cues  • Does not wake or if roused does not stay awake  • Weak, high-pitched or  continuous cry |
| Respiratory | • Normal breathing | • Nasal flaring  • Tachypnoea:  RR > 50 bpm for 6 – 12 months  RR > 40 bpm for > 12 months  • Oxygen saturation ≤ 95%  in air  • Crackles in the chest | • Grunting  • Tachypnoea:  RR > 60 bpm  • Moderate or severe chest indrawing  • Decreased breathing sounds |
| Circulation | • Normal skin and  eyes  • Moist mucous  membranes | • Tachycardia:  > 160 beats/minute for < 1 yr  > 150 beats/minute for 1–2 yrs  > 140 beats/minute for 2–5 yrs  • CRT ≥ 3 seconds  • Dry mucous membranes  • Poor feeding in infants  • Reduced urine output | • Reduced skin turgor |
| Other | • None of the amber  or red symptoms or signs | • Fever for > 5 days  • Rigors  • Swelling of a limb or joint  • Non-weight bearing limb or not using an extremity | • Non-blanching rash  • Bulging fontanelle  • Neck stiffness  • Status epilepticus  • Focal neurological signs  • Focal seizures |

**Supplementary table 1** **Green, amber and red flags indicating risk for severe infection**
Traffic light system with clinical signs and symptoms indicating risk for severe infection as defined by the Dutch FWS guideline, derived from the NICE guideline. Abbreviations: bpm, breaths per minute; CRT, capillary refill time; RR, respiratory rate; yr, year.

| **Clinical criteria** | born at term (≥ 37 weeks gestation) |
| --- | --- |
|  | no treatment for unexplained hyperbilirubinemia |
|  | no previous antimicrobial therapy |
|  | no chronic or underlying illness |
|  | no previous hospitalization |
|  | well appearing (regarding feeding, activity, alertness, tone, peripheral circulation, breathing) |
|  | no evidence of skin, soft-tissue, bone, joint, or ear infection |
| **Laboratory criteria** | white blood cell count of 5 – 15 × 10^9^/L |
|  | absolute band count of < 1,5 × 10^9^/L |
|  | platelet count of > 150 × 10^9^/L |
|  | urinalysis white blood cells <10 per field |
|  | urine leukocyte esterase negative |
|  | urine nitrate negative |

**Supplementary table 2 Rochester criteria for low risk of severe infection**
Rochester criteria as defined by the Dutch FWS guideline.

|  | **Item No** | **Recommendation** | **Page No** |
| --- | --- | --- | --- |
| **Title and abstract** | 1 | (*a*) Indicate the study’s design with a commonly used term in the title or the abstract | 2 |
|  |  | (*b*) Provide in the abstract an informative and balanced summary of what was done and what was found | 2 |
| **Introduction** | | | |
| Background/rationale | 2 | Explain the scientific background and rationale for the investigation being reported | 3,5 |
| Objectives | 3 | State specific objectives, including any prespecified hypotheses | 6 |
| **Methods** | | | |
| Study design | 4 | Present key elements of study design early in the paper | 6,7,8 |
| Setting | 5 | Describe the setting, locations, and relevant dates, including periods of recruitment, exposure, follow-up, and data collection | 6 |
| Participants | 6 | (*a*) Give the eligibility criteria, and the sources and methods of selection of participants | 6 |
| Variables | 7 | Clearly define all outcomes, exposures, predictors, potential confounders, and effect modifiers. Give diagnostic criteria, if applicable | 7, suppl mat |
| Data sources/ measurement | 8* | For each variable of interest, give sources of data and details of methods of assessment (measurement). Describe comparability of assessment methods if there is more than one group | 7 |
| Bias | 9 | Describe any efforts to address potential sources of bias | 9 |
| Study size | 10 | Explain how the study size was arrived at | 9 |
| Quantitative variables | 11 | Explain how quantitative variables were handled in the analyses. If applicable, describe which groupings were chosen and why | 8,9 |
| Statistical methods | 12 | (*a*) Describe all statistical methods, including those used to control for confounding | 9 |
|  |  | (*b*) Describe any methods used to examine subgroups and interactions | 8,9 |
|  |  | (*c*) Explain how missing data were addressed | 8 |
|  |  | (*d*) If applicable, describe analytical methods taking account of sampling strategy | - |
|  |  | (*e*) Describe any sensitivity analyses | - |
| **Results** | | | |
| Participants | 13* | (a) Report numbers of individuals at each stage of study—eg numbers potentially eligible, examined for eligibility, confirmed eligible, included in the study, completing follow-up, and analysed | 9,10 |
|  |  | (b) Give reasons for non-participation at each stage | Fig 2 |
|  |  | (c) Consider use of a flow diagram | Fig 2 |
| Descriptive data | 14* | (a) Give characteristics of study participants (eg demographic, clinical, social) and information on exposures and potential confounders | Table 1 |
|  |  | (b) Indicate number of participants with missing data for each variable of interest | Table 1 |
| Outcome data | 15* | Report numbers of outcome events or summary measures | 10 |
| Main results | 16 | (*a*) Give unadjusted estimates and, if applicable, confounder-adjusted estimates and their precision (eg, 95% confidence interval). Make clear which confounders were adjusted for and why they were included | 10,11 |
|  |  | (*b*) Report category boundaries when continuous variables were categorized | Table 1 |
|  |  | (*c*) If relevant, consider translating estimates of relative risk into absolute risk for a meaningful time period | - |
| Other analyses | 17 | Report other analyses done—eg analyses of subgroups and interactions, and sensitivity analyses | 11 |
| **Discussion** | | | |
| Key results | 18 | Summarise key results with reference to study objectives | 12 |
| Limitations | 19 | Discuss limitations of the study, taking into account sources of potential bias or imprecision. Discuss both direction and magnitude of any potential bias | 14 |
| Interpretation | 20 | Give a cautious overall interpretation of results considering objectives, limitations, multiplicity of analyses, results from similar studies, and other relevant evidence | 14,15 |
| Generalisability | 21 | Discuss the generalisability (external validity) of the study results | 13,14 |
| **Other information** | | | |
| Funding | 22 | Give the source of funding and the role of the funders for the present study and, if applicable, for the original study on which the present article is based | 18 |

*Give information separately for exposed and unexposed groups.

**Supplementary table 3 STROBE reporting checklist**

STROBE Statement—Checklist of items that should be included in reports of ***cross-sectional studie***

| Diagnosis | Criteria in diagnostic test | Clinical signs |
| --- | --- | --- |
| Herpes Simplex virus (HSV) encephalitis | Positive polymerase chain reaction (PCR) with HSV type 1 or 2 on cerebral spinal fluid (CSF) or neurological symptoms and a positive PCR on any sample |  |
| Bacteremia | Positive blood culture with bacteria, excluding probable contaminants |  |
| Bacterial meningitis | Positive CSF fluid with bacteria, excluding probable contaminants |  |
| Urinary tract infection | Positive urine culture, excluding probable contaminants |  |
| Septic Arthritis | Positive culture of joint fluid or a leukocytes count in the joint fluid of >50x10^9^/L |  |
| Osteomyelitis | Abnormal histopathologic examination of bone biopsy, positive tissue culture or abnormalities on MRI (bone marrow oedema or the presence of abscesses) |  |
| Pneumonia | Positive chest X-ray showing consolidations suggestive for infection | Tachypnea with pulmatory ausculatory localized abnormalities (crackles or decreased lung sounds) |
| Kawasaki disease |  | Fever >5 days and at least 4 of the following symptoms:   - Bilateral conjunctivitis - Cervical lymphadenopathy - Mucosal changes - Extremities changes - Polymorphous rash |

**Supplementary table 4 Diagnostic criteria**

| **Site nr** | **Site 1** | **Site 2** | **Site 3** | **Site 4** | **Site 5** | **Site 6** | **Site 7** |
| --- | --- | --- | --- | --- | --- | --- | --- |
| **Inclusions** | 24 | 72 | 78 | 47 | 57 | 40 | 15 |
| **Adherence** | 13 (54%) | 30 (42%) | 30 (39%) | 29 (62%) | 36 (63%) | 20 (50%) | 9 (60%) |
| **Age group**  < 1m  1 – 3 m  > 3m | 9 (38%) 8 (33%) 7 (29%) | 31 (43%)  31 (43%)  10 (14%) | 17 (22%)  35 (45%)  26 (33%) | 23 (49%)  22 (47%)  2 (4%) | 9 (16%)  21 (37%)  27 (47%) | 11 (28%)  22 (55%)  7 (18%) | 1 (7%)  2 (13%)  12 (80%) |
| **Risk group**  Low  Intermediate  High | 6 (29%) 3 (14%) 12 (57%) | 11 (16%) 14 (20%) 45 (64%) | 13 (18%) 13 (18%) 46 (64%) | 5 (11%) 3 (7%) 38 (83%) | 18 (34%) 12 (23%) 23 (43%) | 12 (30%) 10 (25%) 18 (45%) | 8 (53%) 2 (13%)  5 (33%) |
| **Ill appearance** | 4 (17%) | 15 (21%) | 15 (19%) | 16 (34%) | 8 (14%) | 4 (10%) | 5 (33%) |

**Supplementary table 5 Characteristics per site**
